# Legacy effects of cover cropping and crop phase on soybean health and associated rhizosphere microbiome in corn-soybean rotation

**DOI:** 10.1186/s40793-026-00899-3

**Published:** 2026-05-02

**Authors:** Chuntao Yin, Shannon L. Osborne, R. Michael Lehman

**Affiliations:** North Central Agricultural Research Laboratory, USDA-ARS, Brookings, SD USA

**Keywords:** Cover cropping, Crop phase, Soilborne disease, Rhizosphere microbiome, Suppression

## Abstract

**Background:**

Crop diversification through crop rotation or cover cropping is widely recognized as an important strategy to improve agroecosystem sustainability, enhance soil health, and suppress soilborne diseases. Rotating crops or introducing cover crops can disrupt pathogen life cycles, improve nutrient cycling, and promote beneficial microbes. However, the outcomes of diversification practices are often complex, influenced by soil type, crops, and pathogen pressures. Evaluating how cover crops and crop phase affect crop soilborne diseases and root-associated microbiome is critical for designing resilient cropping systems.

**Results:**

This study evaluated the legacy effects of cover crops and crop phase on soybean root diseases and root-associated microbiome. Soybean plants were grown in soils collected from a corn-soybean rotation field experiment with and without cover crops, and then challenged with either *Fusarium graminearum* inoculum or soybean cyst nematode (SCN) in the growth chamber. Soils with a cover crop history significantly reduced *F. graminearum*-induced root rot, but had a limited impact on SCN, indicating divergent disease responses. Microbial profiling revealed that *F. graminearum* inoculum strongly reshaped bacterial communities, reducing Shannon diversity and enriching fast-growing copiotrophic taxa, including Bacteroidota genera (*Pedobacter*, *Chitinophaga*, *Flavobacterium*, and *Mucilaginibacter*) and Proteobacteria genera (*Dyella*, *Pseudomonas*, *Rhizobium*, and *Paraburkholderia*) regardless of cover crops. In contrast, SCN infection increased bacterial Shannon diversity in soybean-phase soils regardless of cover crops but decreased fungal Shannon diversity in soybean soils without cover crops, highlighting pathogen-specific microbial shifts. Cover cropping enhanced microbial heterogeneity under both pathogen pressures, enriching microbial taxa potentially involved in nutrient cycling (*Chitinophaga* and *Mucilaginibacter*), antagonism (*Flavobacterium*,* Streptomyces*, *Pseudonocardia*, and *Nocardioides*), and competitive interactions (*Paraburkholderia*). Correlation analyses further linked specific bacterial and fungal genera with disease suppression.

**Conclusions:**

Soilborne pathogens and cropping practices exerted interconnected, pathogen- and crop-specific effects on root microbial communities. Cover cropping offers a promising strategy to enhance microbial-mediated disease resilience in soybean systems, providing ecological insights into microbiome-driven plant health.

**Supplementary Information:**

The online version contains supplementary material available at 10.1186/s40793-026-00899-3.

## Background

Corn (*Zea mays* L.) and soybean [*Glycine max* (L.) Merr.] dominate agricultural production in the U.S. Midwest [1], accounting for nearly 50% of national cash receipts in 2023 [2]. However, intensive corn-soybean rotations often contribute to soil degradation and environmental stress. Integrating cover crops into this rotation has been recognized as a promising conservation practice to diversify cropping systems and mitigate the negative effects of simplified rotations [3, 4]. Cover crops provide multiple ecosystem services that enhance both soil health and system sustainability. Cover crops prevent soil erosion [5, 6], improve water infiltration and moisture retention [7], and reduce nutrient loss [8, 9]. In addition, cover crops improve soil properties by increasing soil organic carbon [10, 11], promoting aggregation [12], and enhancing soil structure [13, 14]. Biologically, cover crops can suppress weed pressure [15, 16] or reduce certain soilborne plant diseases [17, 18, 19]. As a result of these agronomic and ecological benefits, cover crop adoption in the U.S. increased from 4.2 million ha in 2012 to 6.3 million ha in 2017 [20, 21]. However, responses of subsequent cash crops to cover cropping varied across environments, management practices, and crop rotations, with negative, neutral, or positive outcomes [17, 22, 23].

Crop diversification alters soil microbial structure and function, with diverse rotations generally increasing microbial diversity, functional redundancy, and the soil’s natural disease suppressive capacity [24, 25, 26, 27]. Cover crops can be introduced to increase crop diversity and have been found to further enhance these benefits by enriching beneficial taxa, improving nutrient cycling, and suppressing soilborne pathogens through multiple mechanisms, including root exudation during active growth [28], and longer-lasting influences such as residue inputs, changes in soil organic matter, and alterations in soil moisture and nutrient dynamics following crop termination [29]. For instance, cruciferous cover crops like rape, canola, radish, *Brassica* spp., and arugula released isothiocyanates during decomposition that act as natural biofumigants, effectively suppressing soilborne pathogens [30, 31] and nematodes [29, 32]. However, cover crops do not always provide consistent benefits. For example, cereal rye inhibited *Rhizoctonia solani* in some trials [33] but displayed limited impact on other pathogens [34]. In some cases, cover crops increased pathogen inoculum, enhanced host survival of soilborne diseases, or served as alternative hosts for pests [34, 35]. These effects can be further modulated by the identity of the subsequent cash crops. Corn and soybean imposed distinct rhizosphere selection pressures: soybean enriches symbiotic and N-cycling microbes such as *Bradyrhizobium*, *Rhizobium*, and arbuscular mycorrhizal fungi, while corn favors decomposers linked to residue breakdown and carbon cycling [36, 37]. Together, cover crops and crop phase have the potential to reshape microbial community assembly in ways that promote soil ecosystem balance, enhance soil quality, and reduce pest and disease pressures, but the outcomes remain inconsistent. Therefore, the interactive legacy effects of cover crops and crop phase on soil microbiomes and soil health need further investigation.

This study evaluated the soil legacy effects of five years of cover crop integration and crop phase on soybean health and root-associated microbiomes under controlled conditions. Susceptible soybean was grown in soils collected from corn-soybean rotation plots with and without cover crops, and then challenged with either *Fusarium graminearum* inoculum or soybean cyst nematode (SCN). Plant health and rhizosphere microbial communities were measured to address two objectives: (i) assessing how cover crop history and crop phase influence soybean disease resistance, and (ii) determining whether these effects are linked to shifts in root-associated microbiome. We hypothesized that soils with a history of cover cropping would improve soybean resistance to soilborne pathogens via microbiome-mediated mechanisms. This study provides insights into how cover cropping and crop phase can enhance soil and plant health and shape soil microbiomes, supporting more resilient and sustainable agroecosystems.

## Methods

### Field site, soil collection, and plant

A long-term corn-soybean rotation trial was established in 2001 at the Eastern South Dakota Soil and Water Research Farm in Brookings, SD (44°19′ N, 96°46′ W) [38]. The soil at the site is a Barnes clay loam (fine-loamy, mixed, superactive, frigid calcic Hapludoll). Soilborne pathogens, *F. graminearum* and SCN, were not detectable in the field plots [37]. The experimental design was a randomized complete block with four replications and a plot size of 90 m². Treatments consisted of a two-year corn (Pioneer P9211) -soybean (Pioneer P16T58E) rotation with and without cover crops, with each cash crop present each year. All plots were under a no-till practice. Cover crops were introduced in spring 2017 and interseeded into standing corn (growth stage V7) and soybean (R1) [39]. Winter rye (*Secale cereale* L.) was interseeded into corn at 45 kg ha⁻¹, while a mixture of winter rye, forage turnip (*Brassica rapa*), and clovers-Balansa (*Trifolium michelianum* L.), Berseem (*T. alexandrinum* L.), and Crimson (*T. incarnatum* L.)-was interseeded into soybean at 34 kg ha⁻¹. Cover crops were allowed to grow through the remainder of the growing season, fall, and into the following spring, after which the overwintering species (rye and clovers) were chemically terminated within one day prior to planting corn by applying a mixture of 3,6-dichloro-o-anisic acid (0.84 kg a.i. ha^− 1^), saflufenacil (0.50 kg a.i. ha^− 1^) and glyphosate (1.5 kg a.i. ha^− 1^) or soybean by applying a mixture 2,4-Dichlorophenoxyacetic acid, choline salt (0.78 kg a.i. ha^− 1^), saflufenacil (0.50 kg a.i. ha^− 1^) and glyphosate (1.5 kg a.i. ha^− 1^).

Soils were collected from both corn and soybean phases, with and without cover cropping, in fall 2022 after crop harvest and before ground freeze. Six soil cores were sampled at 0–15 cm depth in each plot with the avoidance of edge effects, following an X-shaped pattern and transferred to the lab. The soils from four replicate plots were pooled and mixed thoroughly and sieved through a 5 mm aperture sieve to remove plant debris and rocks, and then stored at 4 °C for further use. Soybean cultivar ‘Williams 82’, which is susceptible to soilborne fungal pathogen *F. graminearum* and SCN was used in this study.

### *Fusarium graminearum* infection

The *F. graminearum* strain used in this study was previously described by Yin et al. [40], and inoculum preparation followed the method of Okello et al. [41]. Briefly, a 7-day-old culture of *F. graminearum* was transferred into a 1000-ml Erlenmeyer flask containing a twice-autoclaved sand-cornmeal mixture (216 g quartz sand, 24 g cornmeal, and 40 ml ddH_2_O) and incubated at room temperature for 3–4 weeks. The resulting inoculum was air-dried overnight in a laminar flow hood and stored at 4 °C until use. For the infection assay, plastic cone-tainers (40 mm diameter × 210 mm length) were filled with 110 g of field soil and moistened with 10 ml of sterile tap water, followed by the addition of 10 g of *F. graminearum* inoculum. Soybean seeds ‘Williams 82’ were surface-sterilized with chlorine gas [25:1 (v/v) 10% sodium hypochlorite and 12 N HCl] for 16 h [42]. One 4-day-old pregerminated seed was planted per cone-tainer and covered with 2 g of sterile vermiculite. An additional 5 ml of sterile tap water was applied to each cone-tainer. Cone-tainers containing soil without fungal inoculum served as controls. All cone-tainers were arranged in a randomized complete block design (RCBD) and incubated in a growth chamber (16-h photoperiod, 22 ± 2 °C). Each cone-tainer received 10 ml of sterile tap water every other day. After two weeks, plants were gently removed. The rhizosphere soil was collected from soybean roots for microbiome analysis [43]. The leftover roots were rinsed under running tap water. Roots were scanned using an Epson Perfection V700 Photo scanner (Epson America, Inc., Los Alamitos, CA), and root rot severity was quantified as the percentage of discolored (brown or black) root length relative to the total root length using WinRHIZO software (Regent Instruments Inc., Canada). Plant parameters, including shoot length and fresh shoot weight, were measured. Each treatment included six replicates.

### Soybean cyst nematode infection

SCN HG type 7 was originally isolated from a soybean field in Brookings, SD, and multiplied on the susceptible cultivar ‘Williams 82’ in a growth chamber as described by Yin et al. [40]. For SCN population increase, field soil was mixed with sterile quartz sand (4030 silica sand, Unimin Minnesota Corp., Le Sueur, MN) and calcined clay (Turface All Sport Pro, Profile Products, Buffalo Grove, IL) at a 1:1:1 (w/w/w) ratio. For the SCN assay, field soil was mixed with sterile quartz sand at a 2:3 (w/w) ratio. Plastic cone-tainers (40 mm diameter × 210 mm length) were filled with 120 g of mixed soil. One 4-day-old pregerminated seed was planted in each cone-tainer and 4000 SCN eggs [44] were added in a 3-cm-deep hole near the soybean seedling. Cone-tainers without SCN inoculation served as controls. All cone-tainers were arranged in a RCBD and maintained in a growth chamber under controlled conditions (28 ± 2 °C, 16-h photoperiod). Each cone-tainer received 10 ml of water daily during the first three weeks and twice daily during the final two weeks. After five weeks, plants were gently uprooted, and adhering soil was shaken off. SCN eggs and second-stage juveniles were extracted from half of each sample (soil and roots) following the method of Shepherd [45], with modifications described by Yin et al. [40], and counted using a Leica DML compound microscope at 100× magnification (Leica Microsystems Inc., Deerfield, IL). The remaining root halves were used to collect rhizosphere soil for microbiome analysis. Plant growth parameters (shoot length and fresh shoot weight) were measured. Each treatment included six replicates.

### Soil DNA extraction and amplicon sequencing of rhizosphere microbiome

DNA was extracted from the rhizosphere soil samples (a total of 96 samples) using the DNeasy PowerSoil kit (Qiagen, Carlsbad, CA) and a bead beater SPEX 1600 MiniG (Spex SamplePrep, Metuchen, NJ) at 1500 Hz for 1 min. Two extraction blanks were included and processed alongside the samples to monitor potential background contamination. DNA concentration was quantified using a Nanodrop spectrophotometer (Thermo Fisher Scientific, Waltham, MA) and sent to the University of Minnesota Genomics Center for amplification, library construction, and Illumina sequencing. Briefly, Bacterial 16 S rRNA genes (V4 region) were amplified using primers 515 F (GTGYCAGCMGCCGCGGTAA) and 806R (GGACTACNVGGGTWTCTAAT) [46], and fungal ITS regions (ITS1) were amplified using primers ITS1F (CTTGGTCATTTAGAGGAAGTAA) and ITS2R (GCTGCGTTCTTCATCGATGC). A two-step dual-indexed PCR was performed, consisting of an initial 25-cycle amplification followed by a 10-cycle indexing PCR on a 1:100 dilution of the first reaction [47, 48]. As part of the standard quality control protocol, mock community was included to assess sequencing performance and potential bias [48]. Amplicons were pooled, size-selected, spiked with 20% PhiX, and sequenced (2 × 300 bp, V3 chemistry) on an Illumina MiSeq platform.

### Sequence processing

Sequence processing was performed using USEARCH (v11) [49, 50] to denoise sequences and define zero-radius operational taxonomic units (zOTUs, 100% identity). Briefly, primer and barcode sequences were removed, and the first 10 and 30 bp for bacterial reads and 20 and 75 bp for fungal reads were trimmed from the forward and reverse reads, respectively. Paired-end reads were merged allowing up to 15 mismatches within the overlap region, with a minimum overlap identity threshold of 80%. This combination ensured that only read pairs with sufficient overall similarity were merged while accommodating sequencing errors in low-quality regions. High-quality reads were obtained by filtering sequences with a maximum expected error rate of 1, removing singletons, and denoising using the UNOISE3 algorithm [51]. One sample with low ITS sequence reads was removed, resulting in a total of 191 sample sequences remained for downstream analysis. Processed reads were then mapped to zOTU representatives to generate a zOTU abundance table. Taxonomic classification was assigned using the SINTAX algorithm with an 80% confidence threshold against the Ribosomal Database Project (v18) [52] for bacterial sequences and the UNITE database (v9) [53] for fungal sequences. Total of 5,587,230 sequence pairs were obtained that mapped to 47,062 zOTUs for bacteria and 5,182,578 sequence pairs were obtained that mapped to 2,031 zOTUs for fungi (Supplemental Table S1). zOTUs identified as non-bacterial or non-fungal were removed, along with those classified as chloroplasts at the order level or mitochondria at the family level, using the decontam package (v1.26.0) [54]. For downstream analyses, samples were subset by pathogen treatment (including inoculated and non-inoculated controls), and zOTU tables were rarefied independently within each group using the rarefy_even_depth function in phyloseq. Rarefaction depths were set to the minimum per group: 16,424 (*F. graminearum*) and 17,282 (SCN) for bacterial reads, and 17,236 (*F. graminearum*) and 20,717 (SCN) for fungal reads.

### Statistical analysis and data visualization

All data analysis was performed in R (v4.5.1). Prior to statistical analysis, data heteroscedasticity was assessed using Levene’s test with car package [55]. The effects of soils collected from field plots and soybean soilborne pathogens (*F. graminearum* or SCN) on soybean root rot severity, SCN populations, and soybean growth parameters were evaluated using analysis of variance (ANOVA), followed by Tukey’s honestly significant difference (HSD) post hoc test (*p* < 0.05). Microbial data analysis was performed in R using vegan (v2.7.1) [56], ggplot2 (v3.5.1) [57], and phyloseq (v1.50.0) [58] packages. Microbial Shannon diversity was calculated using phyloseq and lme4 (v1.1.37) [59], ANOVA and Tukey’s HSD tests were used to evaluate fixed effects (cover crop, crop phase, and pathogen) and random effects (replicates). Nonmetric multidimensional scaling (NMDS) based on Bray-Curtis dissimilarities was used to visualize rhizosphere community structure. PERMANOVA was performed to determine associations between microbial composition and covariates. Differences in microbial phylum abundances among treatments were evaluated using the Kruskal-Wallis test, followed by Dunn’s post hoc test with Benjamini-Hochberg (FDR) correction for multiple comparisons. For each phylum, treatments labeled with the different letters were significantly different (*p* < 0.05). DESeq2 (v1.46.0) [60] was performed to identify microbial taxa, including genera and zOTUs, that differed in the rhizosphere samples among different field soils and between with and without pathogen inoculations using unrarefied zOTU tables. Briefly, the unrarefied zOTU tables were filtered to remove rare taxa (< 10 total sequences). Wald’s test was used to estimate log_2_ fold changes, and *p* values were adjusted for multiple testing using Benjamini-Hochberg false discovery rate (FDR) correction, with *p* < 0.1 considered significant. Relative abundances of differential microbial taxa were visualized using heatmaps. Spearman correlation analyses were performed to identify microbial genera associated with soybean disease parameters (soybean root rot severity or SCN population density). Samples inoculated with *F. graminearum* or SCN were subset and taxa with zero counts were removed. Low-abundance taxa were filtered based on mean relative abundance (> 0.1%) and prevalence (≥ 20% of samples) to reduce sparsity and multiple testing. Remaining taxa were agglomerated to the genus level and transformed using centered log-ratio (CLR). FDR correction was applied for multiple comparisons, with significance thresholds set at FDR < 0.1. Genera showing significant positive or negative correlations were visualized using bar plots generated with ggplot2.

## Results

### Influence of cover cropping and crop phase on soybean root rot and SCN infection

**Fig. 1:**
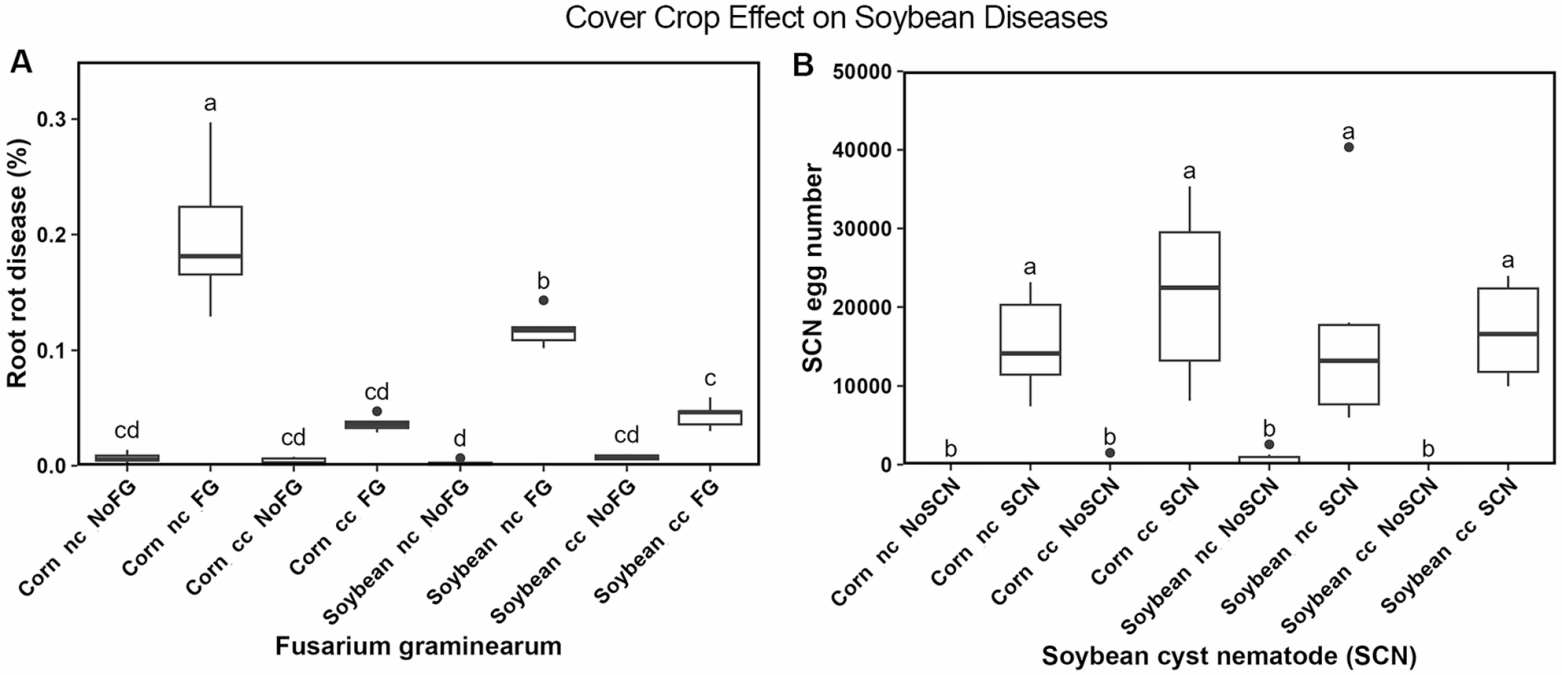
Soil legacy effects of cover cropping and crop phase on soybean soilborne diseases. **A** Root rot caused by *Fusarium graminearum* (root rot severity was quantified as the percentage of diseased root length relative to the total root length). **B** Soybean cyst nematode. Corn: corn phase, soybean: soybean phase, nc: without cover crops, cc: with cover crops, NoFG: without *F. graminearum* inoculum, FG: with *F. graminearum* inoculum, NoSCN: without SCN infection, SCN: with SCN infection. Different letters indicate a statistically significant difference between treatments within an experiment as determined by the Tukey test (*p* < 0.05, *n* = 6 for FG, *n* = 18 for SCN)

Cover cropping and crop phase significantly affected soybean root rot but had a limited effect on SCN infection (Fig. 1). Following *F. graminearum* inoculation, soils from corn phase without cover crops exhibited the highest root rot severity, whereas soils from both corn and soybean phases with cover crops significantly reduced disease incidence compared to without cover crops (*p* < 0.05) (Fig. 1A), indicating that cover cropping enhanced soil suppressiveness to *F. graminearum*, potentially through its interaction with crop phase. Conversely, SCN egg population densities did not differ significantly among SCN inoculation treatments (Fig. 1B), suggesting that neither cover crops nor crop phase influenced SCN infection levels. Additionally, cover cropping, crop phase, and *F. graminearum* inoculation had no significant effects on soybean shoot length or fresh weight (Supplementary Fig. S1A). In contrast, SCN infection slightly reduced soybean shoot growth, with minor variation observed among treatments (Supplementary Fig. S1B).

### Effect of cropping practice and pathogen infection on assembly of bacteria colonizing soybean roots

**Fig. 2:**
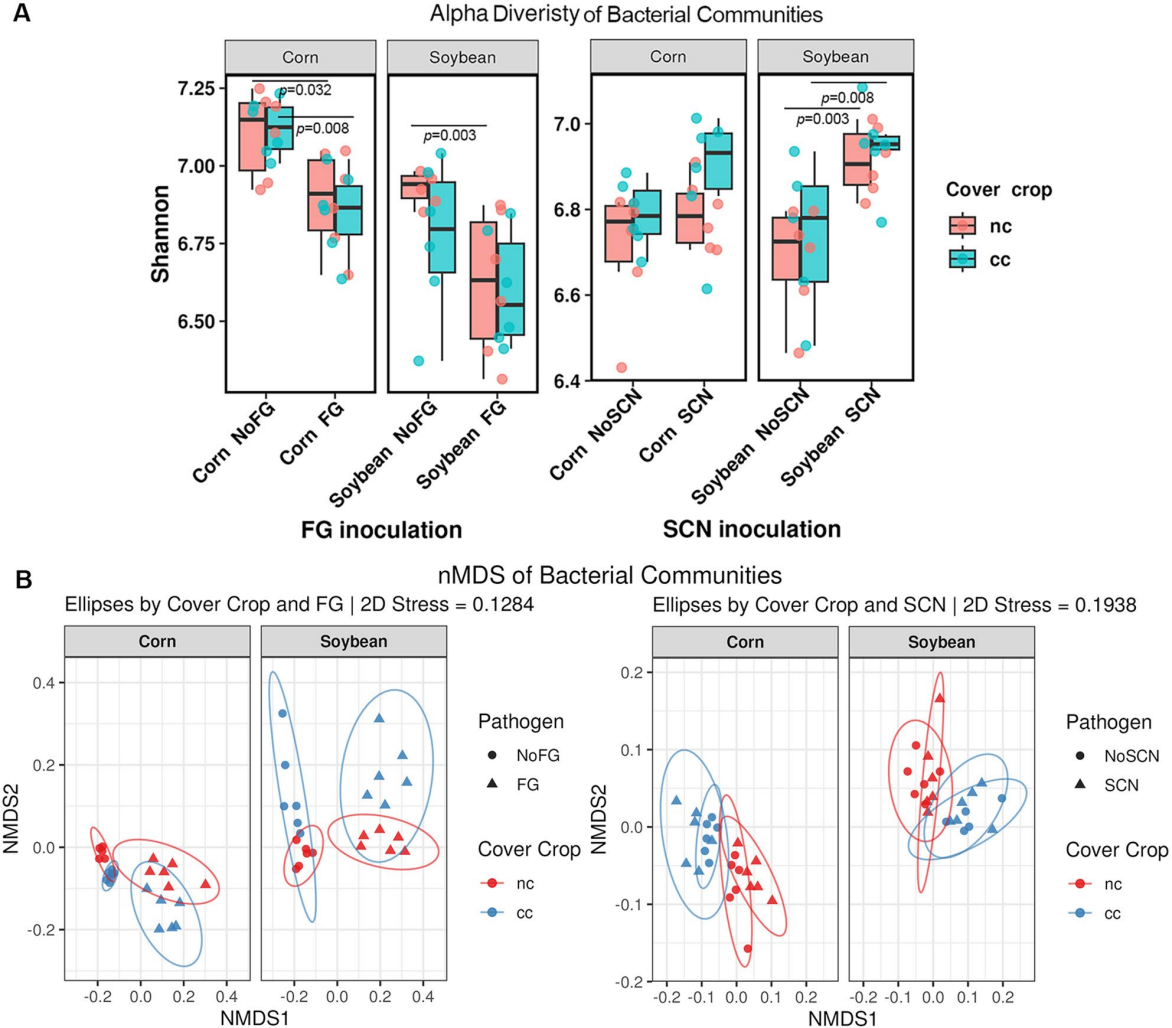
The Shannon diversity and structure of bacterial community in soybean rhizosphere under soilborne pathogen pressures. **A** The Shannon diversity of bacterial community. **B** NMDS ordination of bacterial community. Corn: corn phase, soybean: soybean phase, nc: without cover crops, cc: with cover crops, NoFG: without *F. graminearum* inoculum, FG: with *F. graminearum* inoculum, NoSCN: without SCN infection, SCN: with SCN infection. *p* value: ANOVA and Tukey’s HSD tests

Microbial diversity analysis revealed that pathogen inoculation significantly affected bacterial Shannon diversity, and crop phase also influenced diversity in soybean rhizosphere infested with *F. graminearum* inoculum, whereas cover cropping and their interactions had no significant effects (Supplemental Table S2). *F. graminearum* inoculum significantly reduced Shannon diversity in corn phase soils regardless of cover cropping and in soybean phase soils without cover crops (Fig. 2A). In contrast, SCN inoculation increased Shannon diversity in soybean phase soils, independent of cover cropping, with no similar trend in corn phase soils (Fig. 2A, Supplemental Table S2).

NMDS ordination further demonstrated distinct shifts in bacterial community composition across cover cropping and pathogen treatments (Fig. 2B). Clear separations were observed between *F. graminearum*-inoculated and non-inoculated samples in both corn and soybean phase soils, whereas SCN inoculation produced subtler differences. Cover cropping also contributed to bacterial community variation. PERMANOVA confirmed *F. graminearum* inoculum as the major driver of bacterial compositional changes (*r*² = 0.3505, *p* = 0.001), while cover cropping, crop phase, SCN, and their interactions exhibited smaller but significant effects (*r*² < 0.08, *p* < 0.05) (Supplementary Table S3). Collectively, these results suggested that *F. graminearum* inoculum was the predominant factor shaping bacterial communities, with cropping practices and SCN contributing modest but significant effects.

Across all samples, bacterial communities were dominated by Actinobacteria (31.99% ± 0.63%, mean ± SE), Proteobacteria (31.61% ± 0.50%), Acidobacteria (9.75% ± 0.23%), Thaumarchaeota (6.25% ± 0.22%), and Bacteroidota (6.24% ± 0.46%) (Supplementary Fig. S2). *F. graminearum* inoculum significantly reduced the relative abundances of phyla Acidobacteria, Actinobacteria, and Thaumarchaeota, while increasing Bacteroidetes, regardless of cover cropping and crop phase (Supplemental Fig. S3). SCN decreased Acidobacteria, Actinobacteria, Proteobacteria, and Thaumarchaeota only in corn phase soils with cover crops, with no significant effects in other treatments (Supplemental Fig. S3).

**Fig. 3:**
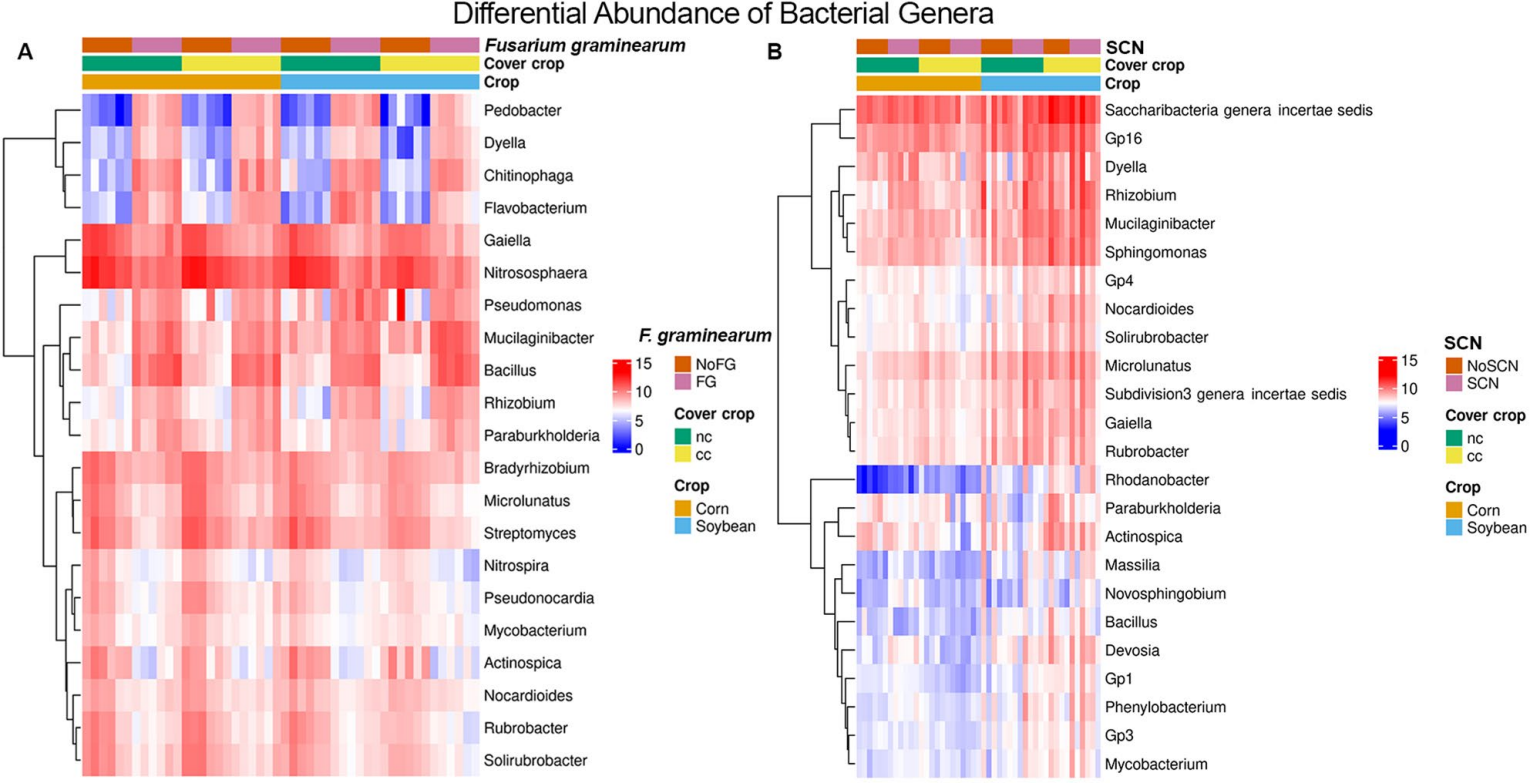
Heatmap of bacterial genera significantly influenced by crop diversification (cover crop and crop phase) and soilborne pathogens (*F. graminearum* or SCN) with a *p* value < 0.05 based on ANOVA of (Log2(1 + *x*))-transformed sequence counts and clustered based on complete-linkage hierarchical clustering of Euclidean distances. Colored bars at the right of the graph presented cropping practice and *F. graminearum* or SCN inoculation for each sample. **A***F. graminearum* inoculum. **B** SCN infection. Corn: corn phase, soybean: soybean phase, nc: without cover crops, cc: with cover crops, NoFG: without *F. graminearum* inoculum, FG: with *F. graminearum* inoculum, NoSCN: without SCN infection, SCN: with SCN infection

At the genus level, *F. graminearum* inoculum consistently increased the relative abundances of Bacteroidetes genera (*Pedobacter*, *Chitinophaga*, *Flavobacterium*, *Mucilaginibacter*), Proteobacteria genera (*Dyella*, *Pseudomonas*, *Rhizobium*, and *Paraburkholderia*), and genus *Bacillus*, while decreasing Actinobacteria genera such as *Gaiella*, *Microlunatus*, *Streptomyces*, *Pseudonocardia*, *Actinospica*, *Nocardioides*, *Rubrobacter*, and *Solirubrobacter* regardless of cropping practices (Fig. 3A). In contrast, SCN and cropping practices exhibited more variable genus-level responses, with no consistent patterns except for enrichment of Proteobacteria genera (*Rhodanobacter*, *Devosia*, *Phenylobacterium*) in soybean phase soils (Fig. 3B).

**Fig. 4:**
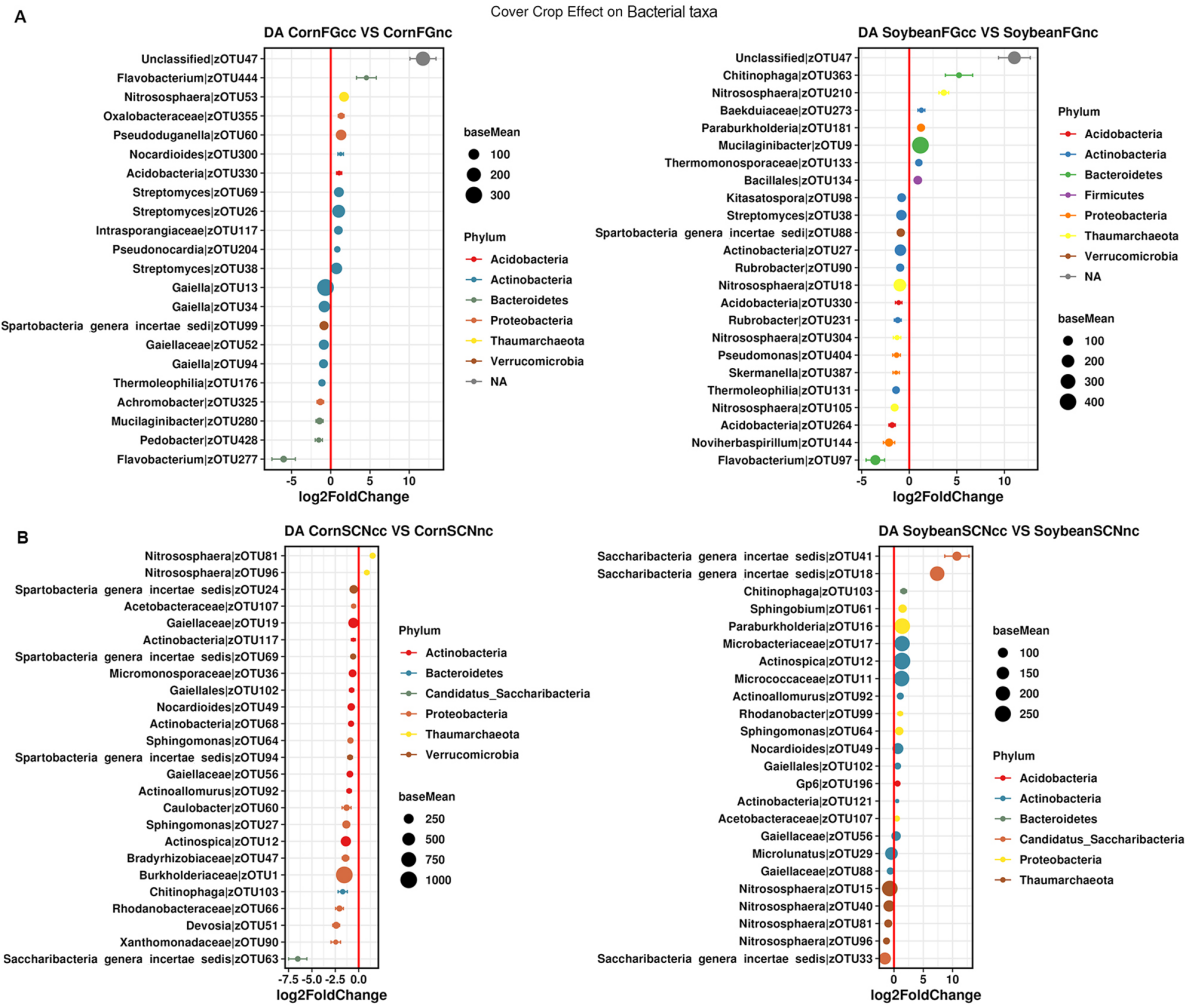
Bacterial zOTUs differed in relative abundance between with cover crop and without cover crop treatments under soilborne pathogen pressures. **A***F. graminearum* inoculum. **B** SCN infection. Values on the *x*-axis presented the DESeq2-estimated log2-fold difference in the soybean rhizosphere grown in the soil with cover crops and without cover crops, where positive values indicate higher abundances in cover crop treatment and negative values indicate reduced abundance without cover crop treatment (FDR adjusted *p*-values of < 0.1 were considered to be differentially abundant, Wald’s test). Dots indicate zOTUs, where the size of the dot is scaled by its mean abundance among all samples (base mean > 100) and its color represents the phylum to which that zOTU belongs. The nearest taxonomy assignment is presented at left. Only zOTUs with a mean abundance > 10 and normalized counts > 5 and present in at least 3 samples were included in the analysis. Corn: corn phase, soybean: soybean phase, nc: without cover crops, cc: with cover crops, FG: with *F. graminearum* inoculum, SCN: with SCN infection

DESeq2 analysis identified bacterial zOTUs responsive to cover cropping under pathogen stress (Fig. 4). In corn phase soils under *F. graminearum* inoculum, zOTUs, classified as genera *Flavobacterium* (zOTU444, Bacteroidetes), *Streptomyces* (zOTU69, zOTU26, and zOTU38), *Pseudonocardia* (zOTU204), *Nocardioides* (zOTU300) (Actinobacteria), and *Nitrososphaera* (zOTU53, Thaumarchaeota) were significantly more abundant in cover cropping than in non-cover crop soils. Similarly, in soybean phase soils with *F. graminearum* inoculum, cover cropping increased zOTUs assigned to *Chitinophaga* (zOTU363, Bacteroidetes), *Paraburkholderia* (zOTU181, Proteobacteria), *Mucilaginibacter* (zOTU9, Bacteroidetes), and *Nitrososphaera* (zOTU210, Thaumarchaeota) (Fig. 4A). Under SCN infection, fewer bacterial taxa (*Nitrososphaera*) (zOTU81 and zOTU96, Thaumarchaeota) were enriched by cover cropping in corn phase soils. In soybean phase soils, however, cover cropping increased zOTUs primarily associated with *Chitinophaga* (zOTU103, Bacteroidetes), *Actinospica* (zOTU12) and *Nocardioides* (zOTU49) (Actinobacteria), and several Proteobacteria genera including *Sphingobium* (zOTU61), *Paraburkholderia* (zOTU16), *Rhodanobacter* (zOTU99), and *Sphingomonas* (zOTU64) (Fig. 4B). Collectively, these results suggested that cover cropping promoted distinct bacterial assemblages under fungal and nematode pathogen pressures.

### Effect of cropping practice and pathogen infection on fungal community in soybean rhizosphere

**Fig. 5:**
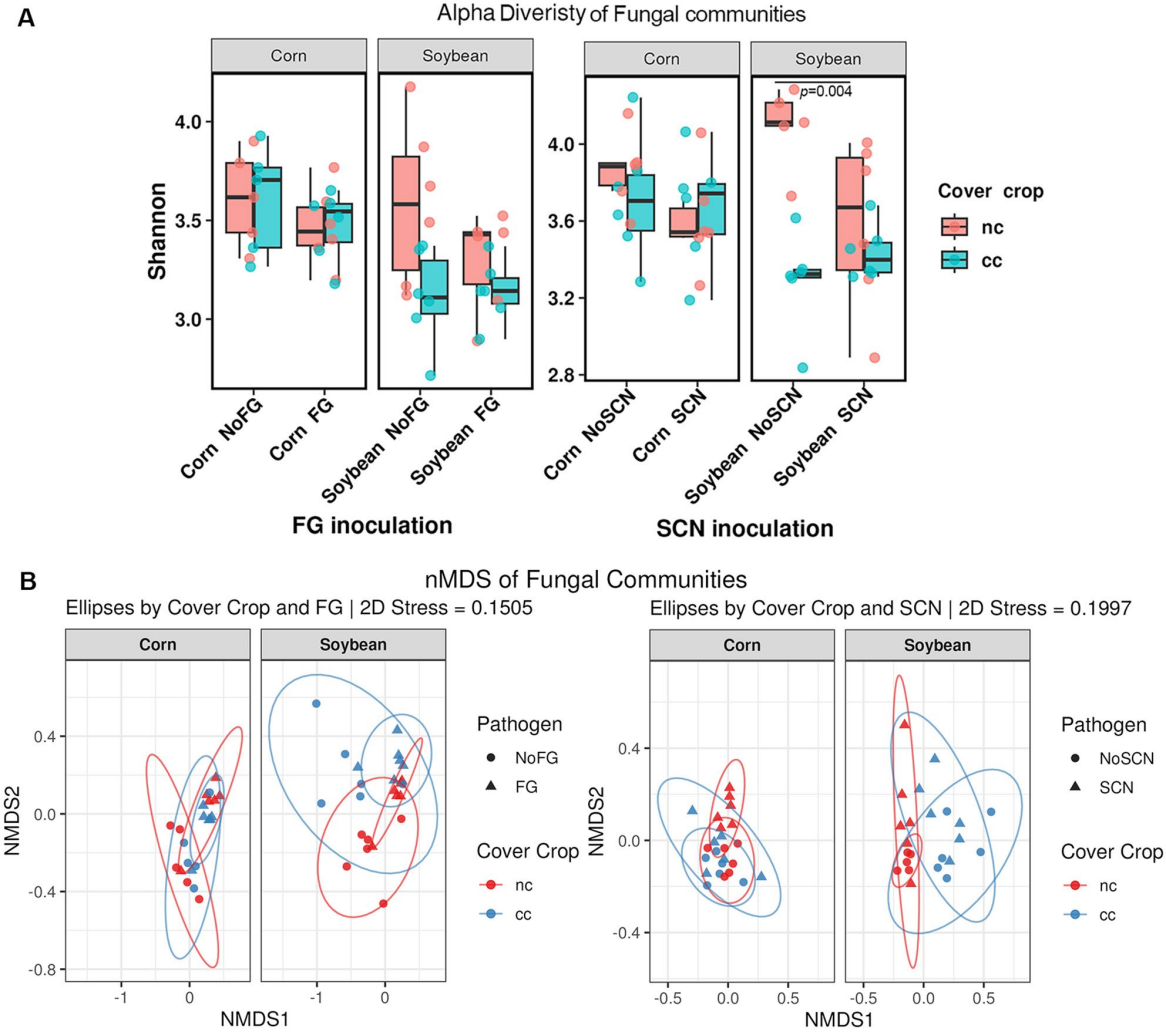
The Shannon diversity and structure of fungal community in soybean rhizosphere under soilborne pathogen pressures. (A) The Shannon diversity of fungal community. (B) NMDS ordination of fungal community. Corn: corn phase, soybean: soybean phase, nc: without cover crops, cc: with cover crops, NoFG: without *F. graminearum* inoculum, FG: with *F. graminearum* inoculum, NoSCN: without SCN infection, SCN: with SCN infection. *p* value: ANOVA and Tukey’s HSD tests

Fungal Shannon diversity was significantly influenced by cover cropping and the interaction between cover crop and crop phase. Moreover, the effects of crop phase and pathogen inoculation on fungal diversity varied under different pathogen infections (Supplementary Table S2). Specifically, SCN inoculation significantly reduced fungal Shannon diversity in soybean phase soils without cover cropping, whereas no significant differences were observed among the other treatments (Fig. 5A). NMDS and PERMANOVA analyses showed that *F. graminearum* inoculation significantly influenced fungal community structure, whereas cropping practices exhibited no clear separation (Fig. 5B, Supplemental Table S3). Similar to bacterial communities, SCN infection and cropping practices (cover cropping and crop phase) showed small but significant effects on fungal community composition (Fig. 5B, Supplemental Table S3).

**Fig. 6:**
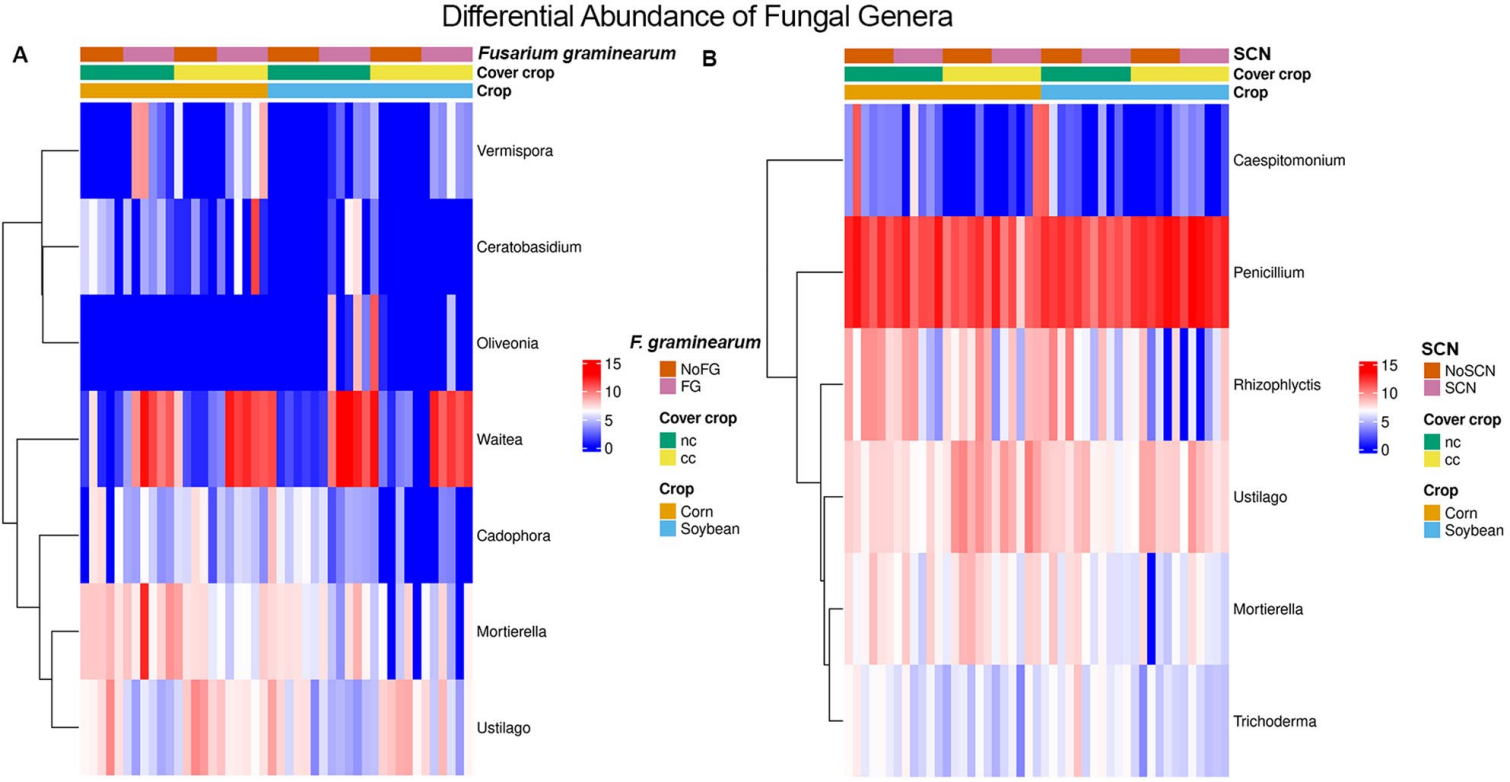
Heatmap of fungal genera significantly influenced by crop diversification (cover crop and crop phase) and soilborne pathogens (*F. graminearum* or SCN) with a *p* value < 0.05 based on ANOVA of (Log2(1 + *x*))-transformed sequence counts and clustered based on complete-linkage hierarchical clustering of Euclidean distances. Colored bars at the right of the graph presented cropping practice and *F. graminearum* or SCN inoculation for each sample. **A***F. graminearum* inoculum. **B** SCN infection. Corn: corn phase, soybean: soybean phase, nc: without cover crops, cc: with cover crops, NoFG: without *F. graminearum* inoculum, FG: with *F. graminearum* inoculum, NoSCN: without SCN infection, SCN: with SCN infection

**Fig. 7:**
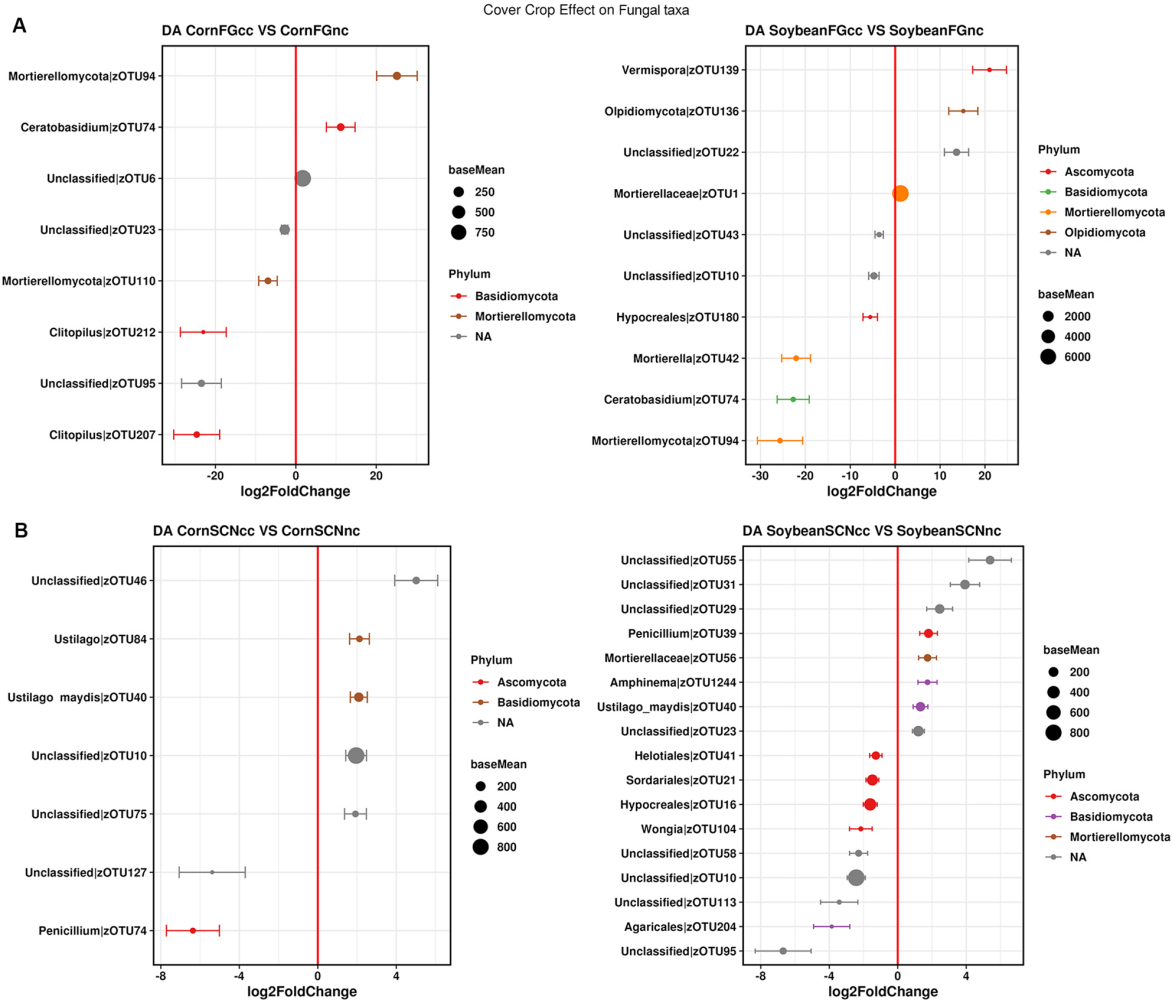
Fungal zOTUs differed in relative abundance between with cover crop and without cover crop treatments under soilborne pathogen pressures. **A***F. graminearum* inoculum. **B** SCN infection. Values on the *x*-axis presented the DESeq2-estimated log2-fold difference in the soybean rhizosphere grown in the soil with cover crops and without cover crops, where positive values indicate higher abundances in cover crop treatment and negative values indicate reduced abundance without cover crop treatment (FDR adjusted *p*-values < 0.1 were considered to be differentially abundant, Wald’s test). Dots indicate zOTUs, where the size of the dot is scaled by its mean abundance among all samples (base mean > 100) and its color represents the phylum to which that zOTU belongs. The nearest taxonomy assignment is presented at left. Only zOTUs with a mean abundance > 10 and normalized counts > 5 and present in at least 3 samples were presented. Corn: corn phase, soybean: soybean phase, nc: without cover crops, cc: with cover crops, FG: with *F. graminearum* inoculum, SCN: with SCN infection

Fungal communities across all samples were dominated by the phyla Basidiomycota (25.49% ± 0.89%), Ascomycota (17.15% ± 0.59%), and Mortierellomycota (3.67% ± 0.23%) (Supplemental Fig. S4). SCN infection significantly decreased the relative abundance of Ascomycota in soybean phase soils without cover cropping, whereas no significant effects on other fungal phyla were observed across the remaining treatments (Supplemental Fig. S5). At the genus level, *F. graminearum* inoculum increased *Vermispora* and *Waitea* regardless of cropping practices (Fig. 6A). SCN and cropping factors showed variable but less pronounced effects (Fig. 6B). Differential abundance analysis further revealed that cover cropping influenced specific fungal zOTUs under pathogen pressure (Fig. 7). Under *F. graminearum* inoculum, cover cropping increased the relative abundance of zOTU94 (Mortierellomycota) and zOTU74 (*Ceratobasidium*) in corn phase soils, and zOTU139 (*Vermispora*) and zOTU136 (Olpidiomycota) in soybean phase soils (Fig. 7A). Under SCN inoculation, cover cropping increased zOTUs classified as *Ustilago maydis* in corn-phase soils and zOTU40 (*Ustilago maydis*), zOTU39 (*Penicllium*), zOTU56 (Mortierellaceae), and zOTU1244 (*Amphinema*) in soybean phase soils, indicating crop- and pathogen-specific fungal responses (Fig. 7B).

### Correlations between microbial genera and soybean diseases

**Fig. 8:**
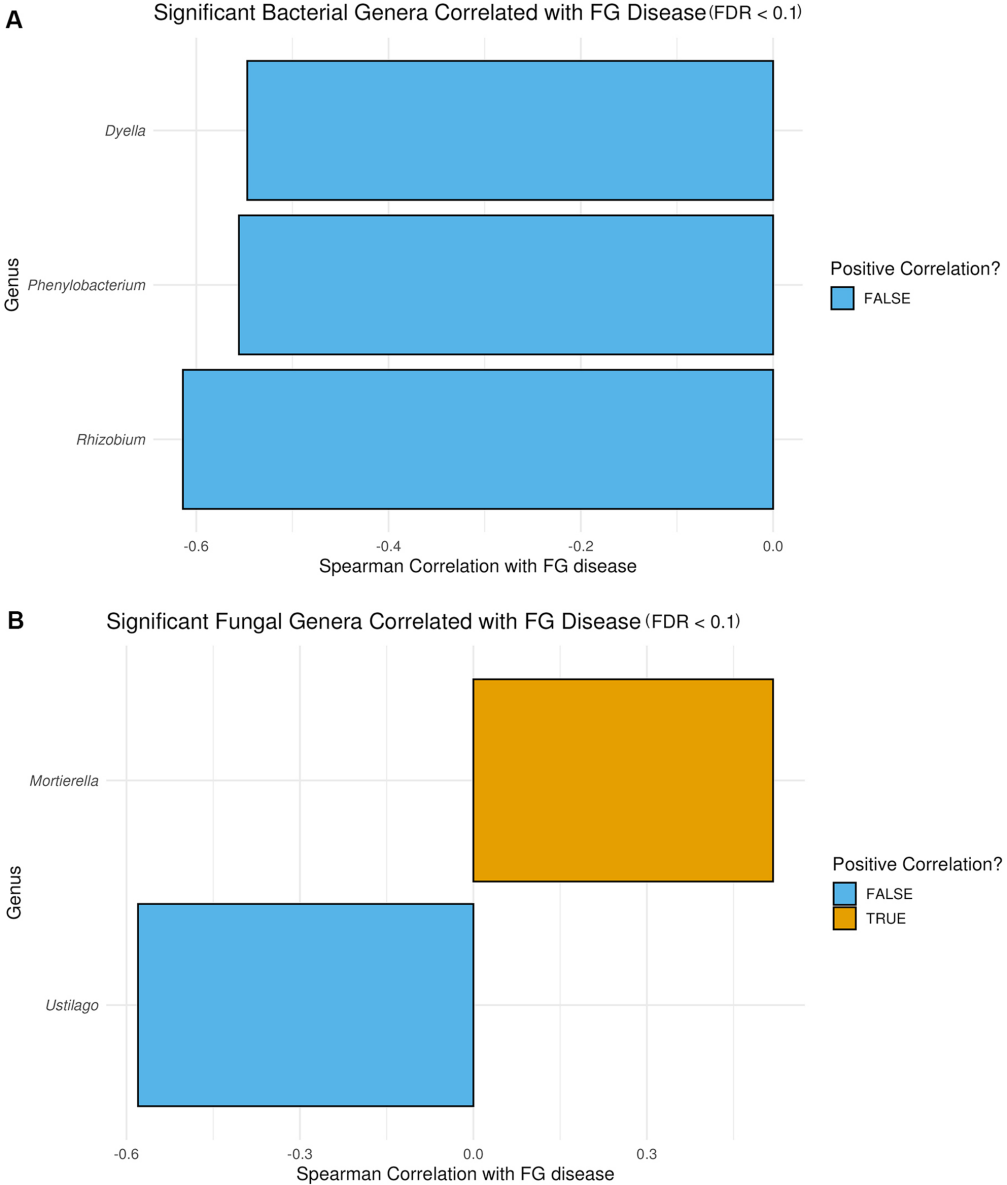
Microbial genera associated with soybean soilborne root rot disease. **A** Bacterial genera. **B** Fungal genera. Blue bar: negative correlation, and yellow bar: positive correlation

Correlation analyses identified microbial genera associations with soybean root rot diseases (Fig. 8). Among bacteria, three genera within phylum Proteobacteria were negatively correlated with soybean root rot disease severity, and they were *Dyella*, *Rhizobium*, and *Phenylobacterium* (Fig. 8A). Two fungal genera were significantly associated with soybean root rot (Fig. 8B). *Mortierella* (Mortierellomycota) showed a positive correlation with root rot disease severity, while *Ustilago* (Basidiomycota) exhibited a negative correlation with root rot. Unlike the patterns observed for *F. graminearum* inoculum, no fungal genera were significantly associated with SCN.

## Discussion

### Effect of cover cropping and crop phase on soybean diseases

Cropping practices are widely adopted as sustainable strategies for soilborne disease management by improving soil properties and disrupting host-pathogen interactions [61, 62]. However, emerging evidence indicates that crop-induced soil legacies may confer complex effects that vary across different cropping systems [63]. In our study, both the soil legacy of cover cropping and crop phase significantly influenced *F. graminearum*-induced soybean root rot. The pronounced reduction in soybean root rot under cover cropping suggested that cover crops could enhance soil suppressiveness by improving soil health [64]. Similar observations have been reported previously: wheat or rye cover crops reduced *Phytophthora* splash dispersal in bell pepper [65]; cereal rye increased soil suppressiveness to *R. solani* and *Fusarium virguliforme* in soybean field trials [33]; and incorporating cover crops reduced *Rhizoctonia* root rot by increasing antagonistic *Pseudomonad* microbial populations [18]. The effects are often linked to increases in organic soil matter and nutrient availability provided by cover crops [66]. The interaction between cover cropping and crop phase likely strengthened this suppressive effect through residue decomposition, as well as changes in soil moisture, nutrient availability, and microbial niches, while the influence of root exudates on microbial recruitment during the growth season may be transient and therefore contribute less to the soil legacy effects observed in this study [63, 64, 67]. Corn phase soils without cover crops showed the highest root rot severity, suggesting a negative effect of corn legacy on soybean root health and aligning with earlier observations that soils preceded by corn caused greater root damage than those preceded by sunflower [68]. This study was conducted as a pot experiment using sieved bulk soil, representing a controlled and disturbed system. Therefore, the results reflected soil legacy effects may differ from in situ field dynamics. In contrast to fungal disease suppression, the limited effect of cover cropping on SCN infection suggested that SCN populations are less responsive to short-term changes in soil organic matter or nutrient inputs, consistent with most recent work showing that SCN establishment is not significantly influenced by cropping history in naïve soils [69]. Although some cover crops have been shown to suppress nematode populations through non-host effects [33, 70, 71] or the production of nematicidal compounds [72, 73], the cover crop species used in this study may not have provided such specific suppression. Together, these results suggested that cover cropping primarily mitigated fungal diseases like *F. graminearum*-induced root rot, while its influence on SCN was limited.

### Effects of cropping practice and pathogen infection on rhizosphere microbial assembly

The plant-associated microbiome plays a pivotal role in enhancing soil health and stress resilience by providing protection against pathogens and supporting disease-suppressive functions [74, 75, 76]. In this study, microbial profiling revealed that soilborne pathogens, cover crops, and crop phase exhibited distinct yet interconnected effects on microbial diversity and community composition in the soybean rhizosphere. Among these factors, *F. graminearum* inoculum emerged as the dominant driver of microbial community shifts, markedly reducing bacterial Shannon diversity and altering the structure and composition of microbial communities. It likely reflected pathogen-induced disequilibrium in the rhizosphere, as *F. graminearum* inoculum selectively suppressed some microbial taxa while favoring others leading to community simplification [77]. Specifically, *F. graminearum* inoculum significantly decreased the relative abundances of phyla Acidobacteria, Actinobacteria, and Thaumarchaeota, while enriching Bacteroidota, indicating a community shift toward fast-growing copiotrophic taxa adapted to pathogen-disturbed environments [78]. The consistent enrichment of Bacteroidota genera such as *Pedobacter*, *Chitinophaga*, *Flavobacterium*, and *Mucilaginibacter*, together with Proteobacteria genera including *Dyella*, *Pseudomonas*, *Rhizobium*, and *Paraburkholderia*, suggested an increased prevalence of microbial groups associated with organic matter degradation and known antifungal capabilities that could influence disease dynamics [79, 80, 81, 82].

In contrast, SCN infection increased bacterial diversity in soybean-phase soils regardless of cover cropping, possibly due to compensatory recruitment of diverse rhizosphere taxa responding to nematode-induced root exudation changes and nutrient leakage [83]. However, SCN infection reduced fungal diversity in soybean soils without cover cropping, indicating that nematode parasitism and the resulting root damage-imposed stress limited fungal proliferation, particularly in systems lacking additional organic inputs from cover crops. These findings aligned with previous studies reporting variable effects of soil-borne pathogens on rhizosphere microbial diversity, highlighting that pathogen impacts are pathosystem-dependent and may differentially affect bacterial and fungal communities within the same pathosystem [75, 77, 81, 83]. SCN infection caused more variable responses, increasing or decreasing specific bacterial groups depending on cover cropping and crop phase, consistent with its indirect effects mediated by nematode-induced nutrient fluxes.

Cover cropping and crop phase also contributed to structuring the microbial community, though their effects were less pronounced than pathogen inoculation. Crop phase likely influenced rhizosphere microbiomes through changes in root exudates, nutrient cycling, and microbial legacies [67]; and cover cropping introduced diverse carbon sources and habitats that supported microbial assemblages [66]. Notably, cover cropping selectively enriched antagonistic, nutrient-competitive, and copiotrophic bacterial zOTUs that inhibit *F. graminearum* through multiple mechanisms: antimicrobial production (*Flavobacterium*,* Streptomyces*, *Pseudonocardia*, and *Nocardioides*) [84, 85, 86], competitive root colonization (*Paraburkholderia*) [87, 88], and enhanced nutrient turnover (*Chitinophaga* and *Mucilaginibacter*) [89, 90]. Under SCN infection, cover cropping increased the relative abundance of a few beneficial bacterial zOTUs classified as *Chitinophaga*,* Nocardioides*, and *Paraburkholderia*, but SCN’s cyst-forming habit may limit beneficial microbes access to its protected life stages and therefore weaken microbial suppression. The enrichment of specific bacterial taxa might partially explain why cover cropping suppressed root rot but not SCN.

Similarly, fungal community responses reflected both soilborne pathogen pressure and crop management effects. *F. graminearum* inoculum increased the relative abundance of *Vermispora* and *Waitea*, consistent with their opportunistic or pathogenic lifestyles [91, 92], while SCN infection reduced *Ascomycota* abundance in low-input (without cover crops) soybean systems, possibly due to nematode-induced root stress. Cover cropping, however, enriched beneficial or saprotrophic fungal groups such as Mortierellomycota [93, 94], *Ceratobasidium*, and *Ustilago maydis*, indicating that additional organic inputs fostered functional fungal guilds. Notably, *Fusarium* was consistently detected at relatively high abundance (≥ 1% at the genus level across samples), but its relative abundance did not differ significantly among treatments. This lack of variation indicated that disease severity was not directly associated with sequencing-based estimates of *Fusarium* abundance, likely due to limited taxonomic resolution, the presence of non-pathogenic *Fusarium* species, and the inability of relative abundance data to reflect absolute pathogen load. Complementary approaches like quantitative PCR can be employed to more accurately quantify specific pathogenic species [39]. Collectively, soilborne pathogens acted as a strong biotic filter driving community shifts, whereas cropping diversification practices promoted microbial heterogeneity, which may contribute to enhanced soil resilience to pathogen invasion.

### Correlations between microbial taxa and soybean diseases

Consistent with previous studies [95, 96], correlation analyses identified several bacterial and fungal genera that were negatively correlated with soybean root rot disease severity, including *Dyella*, *Rhizobium*, *Phenylobacterium*, and *Ustilago*. These taxa were likely linked to healthier rhizosphere and may contribute to disease suppression through complementary mechanisms. For instance, *Rhizobium* is well known for promoting plant growth and exerting biological control through competition with pathogens [97], induction of host systemic resistance [98], and enhanced nutrient acquisition [99]. Consistent with previous findings [100], negative associations between *Dyella* and tomato bacterial wilt caused by *Ralstonia solanacearum* have been reported, and treatment with biocontrol strain *Bacillus cereus* increased *Dyella* abundance in rhizosphere, suggesting a potential role for *Dyella* in disease suppression. Similarly, a lower relative abundance of *Phenylobacterium* was observed in watermelon rhizosphere affected by *Fusarium* [101], and *Phenylobacterium* sp. was positively correlated with suppression of dollar spot disease in turfgrass caused by *Clarireedia jacksonii* [102], supporting our observations. However, an opposite trend was reported in diseased seasame rhizosphere infected by *Ralstonia solanacearum* [103]. The contrasting correlations suggested that *Phenylobacterium* might play divergent roles depending on the host-pathogen system. The decline of *Ustilago*, a potential plant pathogen, under increasing disease pressure may reflect disruption of native fungal assemblages or competitive displacement by pathogenic fungi *F. graminearum* [104]. In contrast, *Mortierella* showed a positive correlation with disease severity, consistent with its role as an opportunistic saprotroph that proliferates in response to root tissue damage and the release of labile carbon during infection [105, 106]. *Mortierella* was also positively correlated with root rot disease in American ginseng [107]. No microbial genera, however, showed significant correlations with SCN populations, suggesting a different mode of host-microbiome interaction compared to fungal pathogens. Unlike necrotrophic fungi, which cause substantial root tissue damage and increase nutrients release into rhizosphere, SCN is a biotrophic parasite that establishes specialized feeding sites within living root cells [108], resulting in more localized and less disruptive alterations to root exudation and rhizosphere chemistry. Accordingly, SCN infection may not strongly restructure the surrounding microbial community at the genus level, limiting detectable microbial associations. Additionally, SCN-microbe interactions are often subtle, strain-specific, or mediated within root tissues rather than community-wide changes, further obscuring correlations. Together, these results indicated that Fusarium root rot drove a pronounced shift from a structured, potentially disease-suppressive microbial community to one dominated by saprotrophic responders, whereas SCN infection exerted a comparatively limited influence on rhizosphere microbial composition. This contrast highlighted the differences in how necrotrophic fungal pathogens and biotrophic nematodes interact with and shape plant-associated microbial communities.

## Conclusions

Cover cropping significantly reduced *F. graminearum*-induced soybean root rot but had minimal impact on SCN infection. *F. graminearum* inoculum was the dominant driver shaping rhizosphere microbial communities, broadly reducing bacterial Shannon diversity while enriching opportunistic, copiotrophic, and predatory taxa adapted to pathogen-stressed soils. Soils with a history of cover cropping likely mitigated root rot by promoting microbial antagonism, nutrient cycling, and competitive interactions, whereas SCN populations remained largely unaffected, likely due to more variable microbial responses and ecological protection. Crop phase contributed secondary and indirect effects by shifting microbial composition rather than providing direct pathogen suppression. These results highlighted the potential of cover cropping to alter rhizosphere microbiomes leading to improved soil health and enhanced resilience against fungal root rot diseases in soybean production systems.

## Supplementary Information

Below is the link to the electronic supplementary material.

Supplementary Material 1. The length and weight of soybean fresh shoot after grown in the field soils challenged with soilborne pathogens.** A** F. graminearum inoculum.** B** SCN infection. Corn: corn phase, soybean: soybean phase, nc: without cover crops, cc: with cover crops, NoFG: without F. graminearum inoculum, FG: with F. graminearum inoculum, NoSCN: without SCN infection, SCN: with SCN infection. Different letters indicate a statistically significant difference between treatments within an experiment as determined by the Tukey test (*p* < 0.05, n = 6).

Supplementary Material 2. Top10 bacterial phyla and their percentage contribution (abundance) in the soybean rhizosphere grouped by treatments across all samples. Corn: corn phase, soybean: soybean phase, nc: without cover crops, cc: with cover crops, NoFG: without *F. graminearum* inoculum, FG: with *F. graminearum* inoculum, NoSCN: without SCN infection, SCN: with SCN infection.

Supplementary Material 3. Bacterial phyla influenced by crop diversification and soilborne pathogen infection. Corn: corn phase, soybean: soybean phase, nc: without cover crops, cc: with cover crops, NoFG: without F. graminearum inoculum, FG: with F. graminearum inoculum, NoSCN: without SCN infection, SCN: with SCN infection. Statistical analysis was within taxa and different letters indicate a statistically significant difference between treatments within an experiment as determined by the Tukey test (*p* < 0.05, n = 6).

Supplementary Material 4. Top10 fungal phyla and their percentage contribution (abundance) in the soybean rhizosphere grouped by treatments across all samples. Corn: corn phase, soybean: soybean phase, nc: without cover crops, cc: with cover crops, NoFG: without F. graminearum inoculum, FG: with F. graminearum inoculum, NoSCN: without SCN infection, SCN: with SCN infection.

Supplementary Material 5. Fungal phyla influenced by crop diversification and soilborne pathogen infection. Corn: corn phase, soybean: soybean phase, nc: without cover crops, cc: with cover crops, NoFG: without F. graminearum inoculum, FG: with F. graminearum inoculum, NoSCN: without SCN infection, SCN: with SCN infection. Statistical analysis was within taxa and different letters indicate a statistically significant difference between treatments within an experiment as determined by the Tukey test (*p* < 0.05, n = 6).

Supplementary Material 6..

## Data Availability

Data from this study is shared in the supplementary material and amplicon sequencing data have been deposited in the NCBI Sequence Read Archive under BioProject accession number PRJNA1369638 (available currently).
