# Supplementary figures and images for: Legacy effects of cover cropping and crop phase on soybean health and associated rhizosphere microbiome in corn-soybean rotation
Source: Environ Microbiome. 2026 May 2;21:80. doi: 10.1186/s40793-026-00899-3 (PMC13285196; doi:10.1186/s40793-026-00899-3)

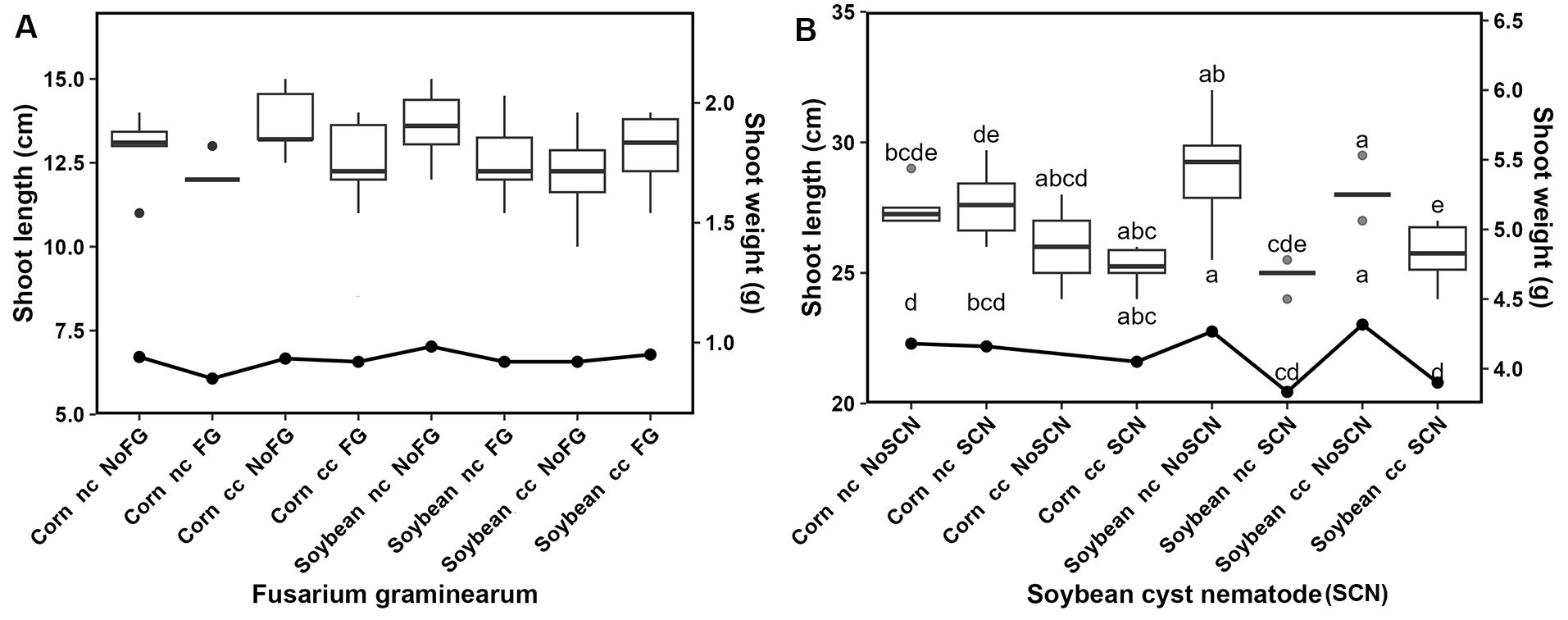

Supplement: Supplementary file 1 — Supplementary Material 1. The length and weight of soybean fresh shoot after grown in the field soils challenged with soilborne pathogens. A F. graminearum inoculum. B SCN infection. Corn: corn phase, soybean: soybean phase, nc: without cover crops, cc: with cover crops, NoFG: without F. graminearum inoculum, FG: with F. graminearum inoculum, NoSCN: without SCN infection, SCN: with SCN infection. Different letters indicate a statistically significant difference between treatments within an experiment as determined by the Tukey test (p < 0.05, n = 6). [file 40793_2026_899_MOESM1_ESM.jpg]

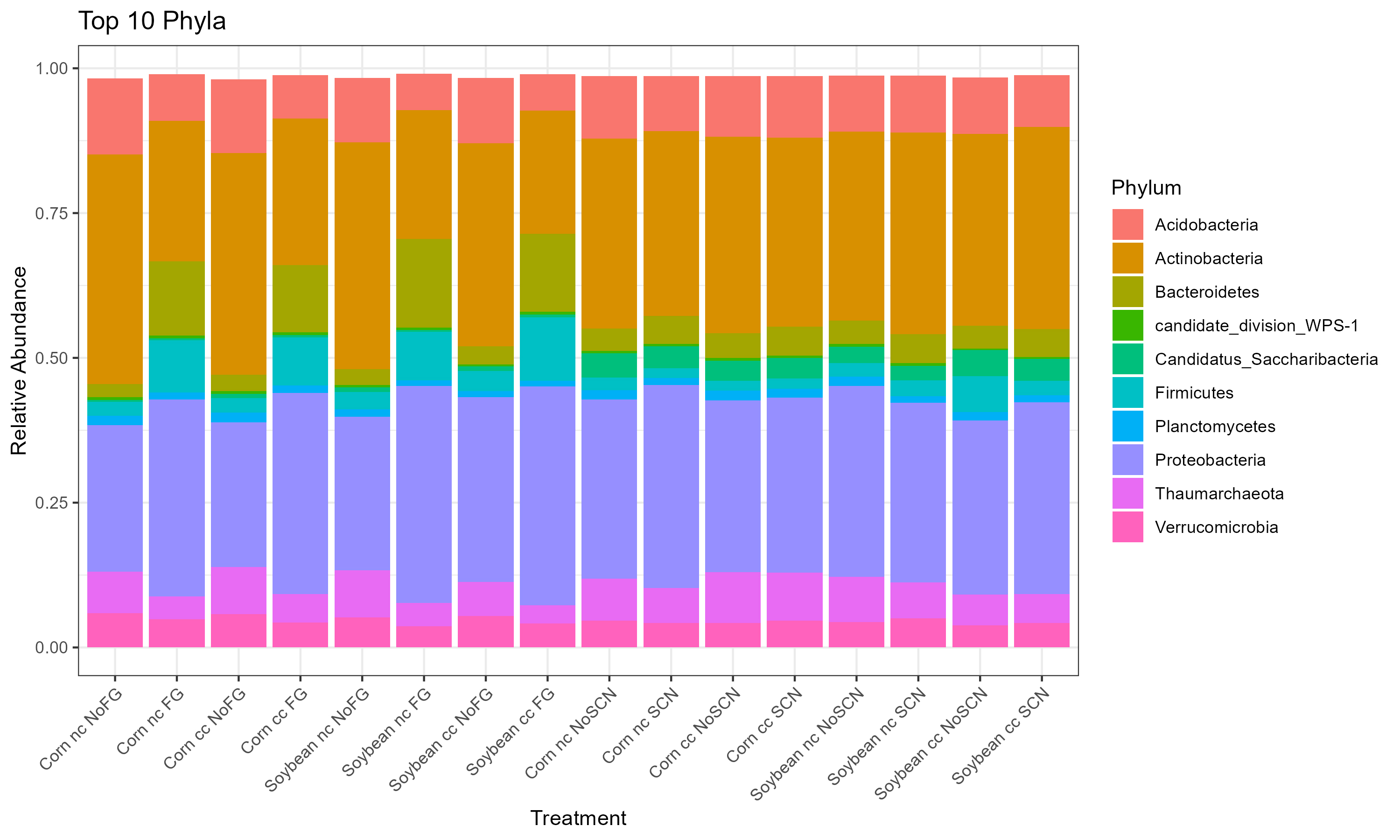

Supplement: Supplementary file 3 — Supplementary Material 3. Bacterial phyla influenced by crop diversification and soilborne pathogen infection. Corn: corn phase, soybean: soybean phase, nc: without cover crops, cc: with cover crops, NoFG: without F. graminearum inoculum, FG: with F. graminearum inoculum, NoSCN: without SCN infection, SCN: with SCN infection. Statistical analysis was within taxa and different letters indicate a statistically significant difference between treatments within an experiment as determined by the Tukey test (p < 0.05, n = 6). [file 40793_2026_899_MOESM3_ESM.jpg]

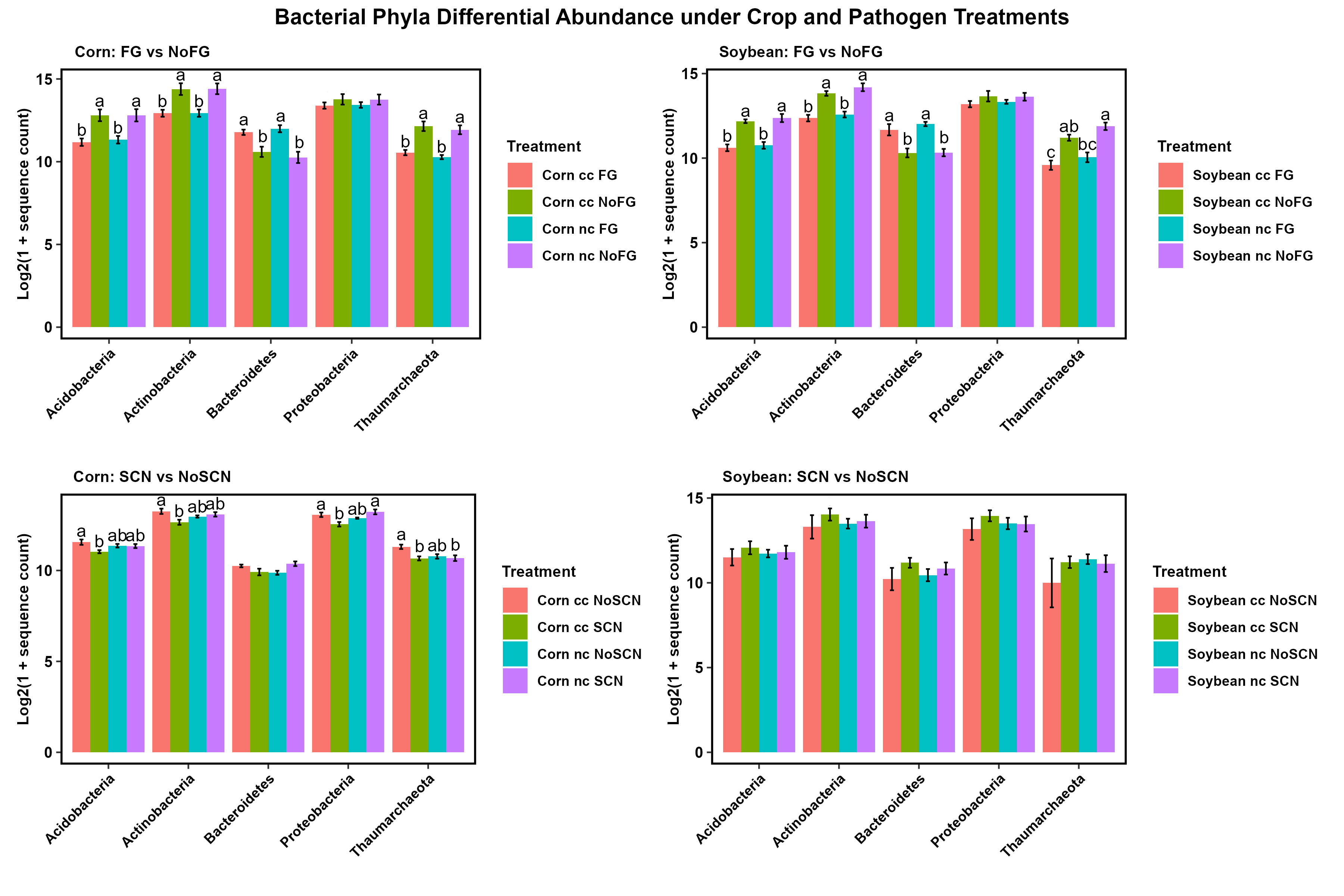

Supplement: Supplementary file 4 — Supplementary Material 4. Top10 fungal phyla and their percentage contribution (abundance) in the soybean rhizosphere grouped by treatments across all samples. Corn: corn phase, soybean: soybean phase, nc: without cover crops, cc: with cover crops, NoFG: without F. graminearum inoculum, FG: with F. graminearum inoculum, NoSCN: without SCN infection, SCN: with SCN infection. [file 40793_2026_899_MOESM4_ESM.jpg]

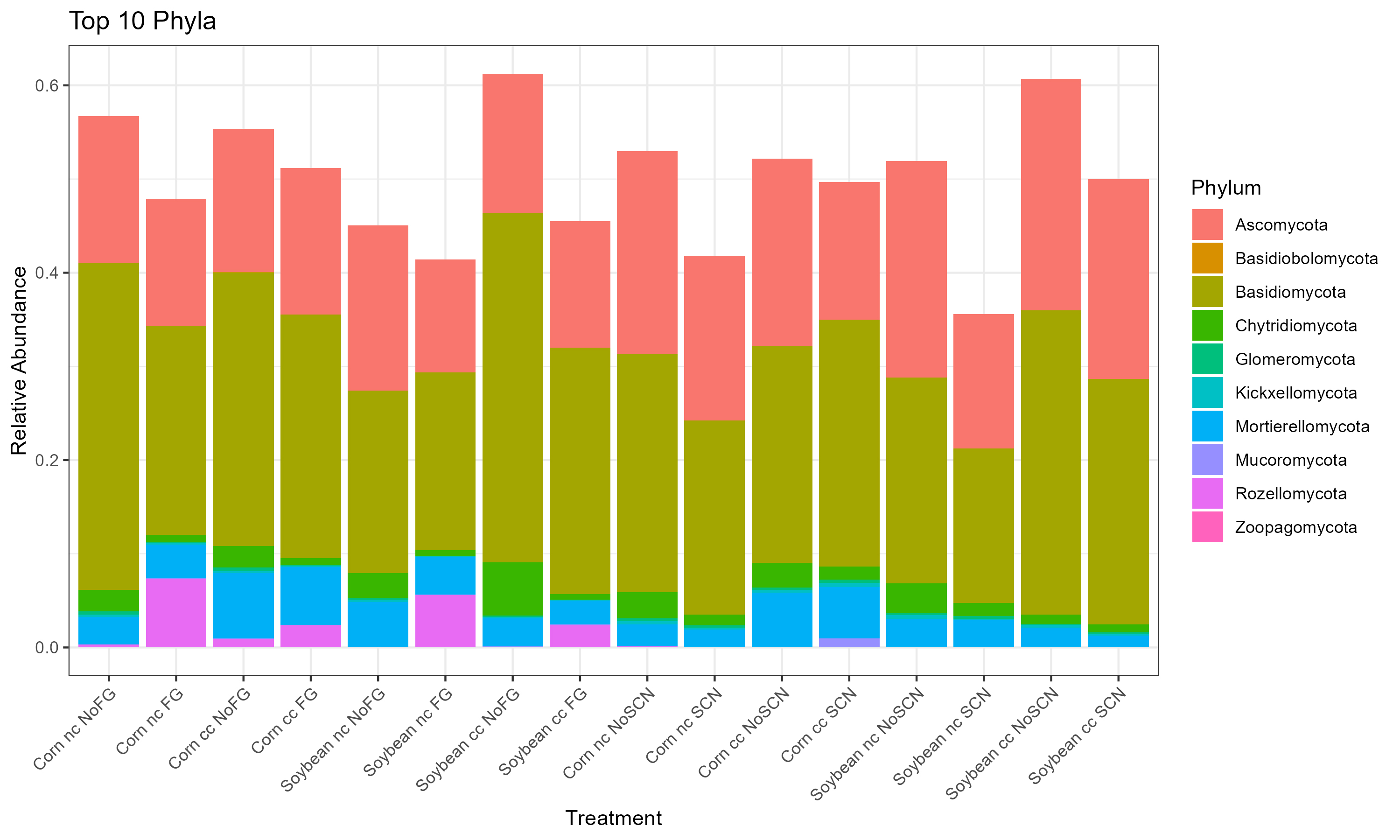

Supplement: Supplementary file 5 — Supplementary Material 5. Fungal phyla influenced by crop diversification and soilborne pathogen infection. Corn: corn phase, soybean: soybean phase, nc: without cover crops, cc: with cover crops, NoFG: without F. graminearum inoculum, FG: with F. graminearum inoculum, NoSCN: without SCN infection, SCN: with SCN infection. Statistical analysis was within taxa and different letters indicate a statistically significant difference between treatments within an experiment as determined by the Tukey test (p < 0.05, n = 6). [file 40793_2026_899_MOESM5_ESM.jpg]

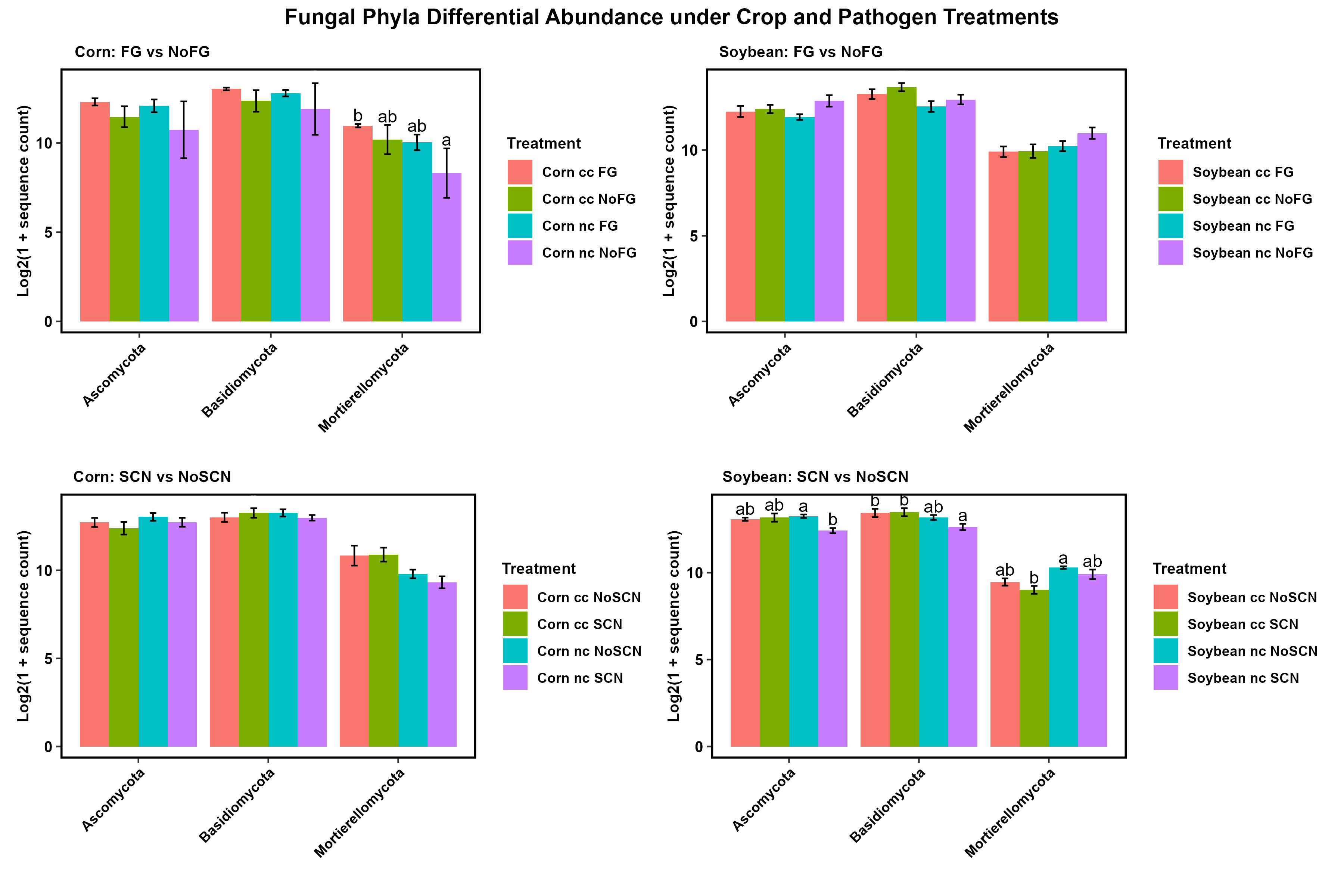

Supplement: Supplementary file 6 — Supplementary Material 6. [file 40793_2026_899_MOESM6_ESM.jpg]
